# Lactylation in diabetes mellitus and its complications: mechanisms of action and therapeutic potential - recent advances

**DOI:** 10.3389/fendo.2025.1710645

**Published:** 2025-11-28

**Authors:** Min Zhou, Lan Liu, Yihong Sun, Xiaoshu Wang

**Affiliations:** Department of Endocrinology, Metabolism and Geriatrics, West China Hospital, Sichuan University, Sichuan University West China Hospital Guang’an Hospital, Guang’an People’s Hospital, Guang’an, Sichuan, China

**Keywords:** lactylation, diabetes mellitus, complications, post-translational modification, metabolic-epigenetic regulation, therapeutic targets

## Abstract

**Background:**

Diabetes mellitus (DM) and its complications represent a global health burden. Lactylation, a novel post-translational modification (PTM) linking metabolism to epigenetic regulation, has emerged as a key mediator in metabolic disorders.

**Objective:**

This review systematically summarizes the regulatory roles and therapeutic potential of lactylation in DM pathogenesis and complications. Key Findings: (1) Lactylation modulates core diabetic pathways by targeting IRS-1 (promoting insulin resistance), NLRP3 (amplifying inflammation), and FOXO1 (enhance oxidative stress); (2) Tissue-specific regulation is observed in complications: lactylation of LARS1 and ACSF2 exacerbates podocyte injury and mitochondrial dysfunction in nephropathy; the FTO-CDK2 axis drives vascular anomalies in retinopathy; and H4K12 lactylation activates Foxo1-mediated oxidative stress in cognitive impairment.

**Conclusion:**

Lactylation functions as a critical metabolic-epigenetic hub, and targeting its “writer-eraser-reader” system may offer novel therapeutic strategies for DM and complications, requiring further clinical translation.

## Graphical Abstract

**Figure:**
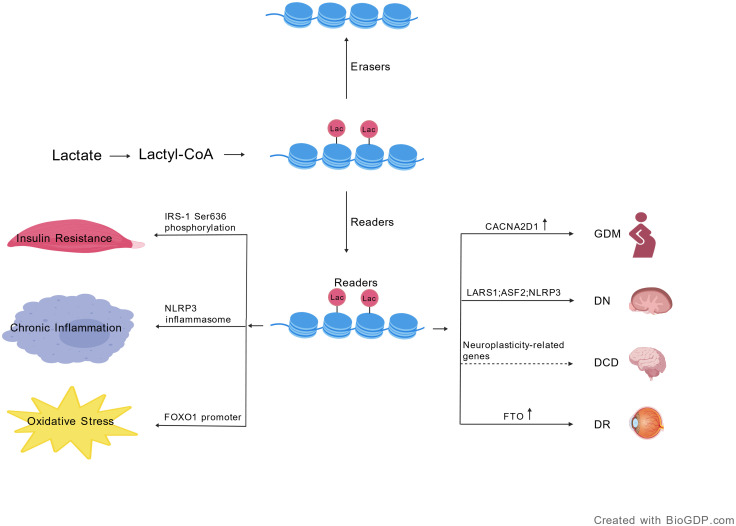
This figure illustrates the dynamic regulatory network of lactylation modification and its core pathological mechanisms in diabetes mellitus and its complications. At the upstream metabolic level, lactate is converted to lactyl-CoA, which mediates lactylation modification (labeled as "Lac") on target proteins (e.g., histones) via enzymatic or non-enzymatic mechanisms. This modification can be reversed by erasers and recognized by readers to exert downstream effects. In diabetic pathogenesis, lactylation contributes to disease progression through key pathways: (1) Promoting IRS-1 Ser636 phosphorylation to exacerbate insulin resistance; (2) Activating the NLRP3 inflammasome to amplify chronic inflammation; (3) Regulating FOXO1 promoter activity to enhance oxidative stress. In tissue-specific complications, lactylation drives disease development via targeted molecular mechanisms: Upregulation of CACNA2D1 in gestational diabetes mellitus (GDM); Lactylation of LARS1 and ACSF2 induces podocyte injury and mitochondrial dysfunction, respectively, in diabetic nephropathy (DN), with NLRP3 inflammasome further aggravating renal fibrosis; Lactylation modulates neuroplasticity-related gene expression in diabetic cognitive dysfunction (DCD); Upregulation of FTO promotes vascular anomalies in diabetic retinopathy (DR). This network reveals lactylation as a critical metabolic-epigenetic hub in multisystem complications of diabetes. Created with BioGDP.com.

## 1 Introduction

Diabetes mellitus (DM) is a chronic metabolic disorder defined by persistent hyperglycemia, poses a significant global health burden due to its escalating prevalence over recent decades. This condition has established itself as a leading contributor to global morbidity and mortality, with its pathophysiology characterized by a complex interplay of insulin resistance, chronic inflammation, and metabolic disturbances that trigger cascading complications affecting multiple organ systems—including the cardiovascular, renal, and nervous systems (1). Within this intricate pathophysiological framework, post-translational modifications (PTMs) have emerged as pivotal regulatory mechanisms, garnering increasing attention for their role in driving diabetes progression and its associated complications (2). A particularly notable PTM in this context is lactylation, a novel modification in which lysine residues on proteins (including histones) undergo covalent attachment of a lactyl group (3).

Recent studies have illuminated the complex interplay between lactate metabolism and lactylation, suggesting that lactate, traditionally viewed as a mere byproduct of glycolysis, plays a significant role in cellular signaling and gene regulation. Lactylation modification participation in the development of various by regulating metabolism, gene transcription, and immune microenvironment: In tumors, it promotes metabolic reprogramming and proliferation, induces immune suppression, and enhances treatment resistance. In metabolic diseases, it promotes lipid deposition in non-alcoholic fatty liver disease and participates in fat accumulation and insulin resistance in obesity and metabolic syndrome. In neurodegenerative diseases, it exacerbates neuroinflammation and microglial dysfunction in Alzheimer’s disease, accelerates the progression of cerebral ischemia/hypoxia diseases, and aggravates nerve injury. In cardiovascular diseases, it reduces myocardial infarction and heart failure injury, delays atherosclerotic plaque formation, and promotes pulmonary hypertension vascular remodeling. In inflammatory and immune diseases, it aggravates systemic inflammatory response in sepsis and participates in abnormal immune tolerance in autoimmune diseases by regulating Treg differentiation. In bone disease, kidney diseases, and eye diseases, it promotes osteogenic differentiation, exacerbates mitochondrial damage and apoptosis, and promotes axial elongation, respectively. Metabolic-epigenetic hub: A molecular node that integrates metabolic signals (e.g., lactate) with epigenetic regulation (e.g., histone modification) to modulate gene expression and cellular phenotypes in metabolic diseases. This paradigm shift holds particular relevance in diabetic pathways and potentiates inflammatory cascades. Lactylation has been linked to various biological processes, including the regulation of gene expression, immune responses, and metabolic homeostasis (4–6). In the context of diabetes, lactylation may contribute to the development of complications such as diabetic nephropathy, retinopathy, and cardiovascular diseases by modulating inflammatory pathways and altering cellular responses to hyperglycemia (7–10).

The mechanistic basis of lactylation in diabetes remains incompletely elucidated; yet accumulating evidence supports its intimate link to metabolic dysregulation. Elevated glucose levels can lead to increased lactate production, which in turn promotes histone lactylation—a modification that can affect gene transcription and cellular function (11). Notably, lactylation has been shown to influence the expression of pro-inflammatory cytokines and other factors that exacerbate tissue damage in diabetic complications (7). Moreover, lactylation may also play a role in the phenomenon of metabolic memory, where prior hyperglycemic episodes continue to influence cellular behavior long after glucose levels have normalized (2). Deciphering these mechanisms is crucial for identifying potential therapeutic targets that could mitigate the long-term effects of diabetes and improve patient outcomes.

Targeting lactylation holds significant therapeutic promise in diabetes management. By modulating lactylation pathways, novel interventions may be developed to not only ameliorate hyperglycemia but also disrupt the molecular underpinnings of diabetic complications. Current studies are exploring various strategies, including the use of lactylation inhibitors and agents that can enhance the body’s ability to manage lactate levels, as potential therapeutic avenues (7, 12, 13). This review aims to systematically explore the role of lactylation in the context of diabetes and its complications, focusing on the molecular mechanisms involved and the prospects for therapeutic interventions. By integrating insights from recent research, we hope to provide a comprehensive overview of how lactylation may serve as a critical link between metabolic dysregulation and the pathophysiology of diabetes, ultimately paving the way for innovative treatment strategies.

## 2 Molecular mechanism of lactylation and its regulatory role in diabetes and complications

### 2.1 Discovery and molecular mechanisms of lactylation

#### 2.1.1 The discovery of lactylation

In 2019, Professor Yingming Zhao’s team first identified the presence of lactylation in core histones of human MCF-7 cells via high-performance liquid chromatography-tandem mass spectrometry (HPLC-MS/MS) analysis, thereby confirming lactylation as a novel epigenetic modification (14). Lactylation modulates gene expression by altering histone conformation, which in turn affects chromatin packaging and accessibility. Studies have demonstrated that lactylation can either activate or repress gene transcription, with its specific effect depending on the modified site and cellular context (15). Furthermore, lactylation can indirectly regulate gene expression by influencing the binding capacity of transcription factors. This pioneering discovery laid a critical foundation for subsequent research (16).

Subsequent studies further validated the universality of lactylation and identified multiple lactylation sites in various cells and tissues, leading to rapid expansion of research in this field. It has been confirmed that lactylation can directly stimulate gene transcription and regulate cellular metabolism and signal transduction (14). In recent years, the role of lactylation in various diseases has been increasingly elucidated, including cancer (17), metabolic diseases (18), inflammatory diseases (14), cardiovascular diseases (19), and neuropsychiatric disorders (20).

#### 2.1.2 Biochemical basis of lactylation modification

Lactylation is primarily facilitated by lactate-derived moieties such as L-lactyl-CoA (L-La-CoA) and lactylglutathione (LGSH), which serve as lactyl donors. The formation of lactylation occurs through both enzymatic and non-enzymatic mechanisms. The enzymatic pathway is predominantly mediated by the acetyltransferase family, with enzymes like YfiQ playing a pivotal role in facilitating the transfer of the lactyl group to target proteins. This process underscores the importance of lactate metabolism in cellular signaling and protein function modulation. Conversely, the non-enzymatic mechanism relies on chemical lactyl group transfer, which can occur under specific physiological conditions, particularly during metabolic stress or hypoxia, leading to increased lactate levels. This dual mechanism of lactylation formation highlights the intricate relationship between metabolic states and protein modifications, suggesting that lactylation may serve as a metabolic sensor, linking energy status to cellular signaling pathways. Recent studies have indicated that lactylation can influence various cellular processes, including gene expression, protein function, and metabolic regulation, thereby establishing its significance in both physiological and pathological contexts, such as cancer and metabolic disorders (21, 22).

#### 2.1.3 “Writers, “ “erasers,“ and “readers” of lactylation

**Figure 1:**
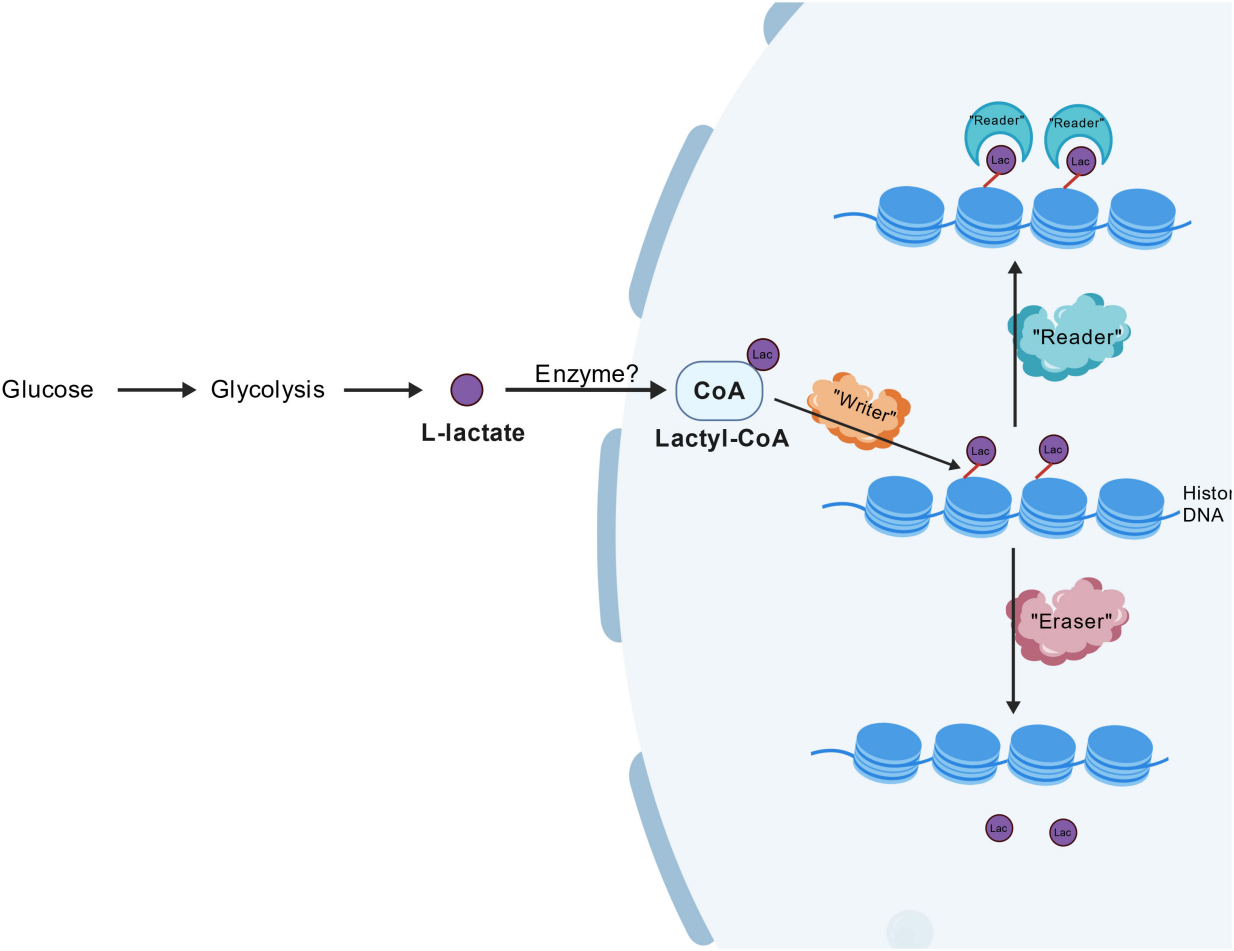
Dynamic regulatory network of lactylation modification. Catalytic addition by writers, reversible removal by erasers, and mechanism of mediating role by potential readers. This figure was created with BioGDP.com (25).

The regulation of lactylation involves specific enzymes known as “writers, “ “erasers, “ and “readers.” The “writers” are primarily acetyltransferases, such as YfiQ, which catalyze the addition of lactyl groups to lysine residues on target proteins, thereby promoting lactylation. On the other hand, “erasers” include deacetylases like SIRT1 and SIRT3, which remove lactyl groups, reversing the modification and potentially restoring the original protein function. This dynamic interplay between lactylation and de-lactylation is crucial for maintaining cellular homeostasis and responding to metabolic changes. While the specific “readers” that recognize lactylated proteins remain largely unidentified, it is hypothesized that specialized proteins may exist to mediate the downstream signaling pathways initiated by lactylation. The identification of these readers is essential for understanding the full spectrum of lactylation’s biological implications, particularly in the context of cellular signaling and gene expression regulation. The complex regulatory network involving writers, erasers, and potential readers underscores the multifaceted role of lactylation in cellular physiology and its potential impact on various diseases, including cancer and metabolic disorders (23, 24). This dynamic regulatory network is illustrated in **Figure 1**.

### 2.2 Lactylation in the pathogenesis of diabetes

#### 2.2.1 Lactylation and insulin resistance

Lactylation, a lactate-induced post-translational modification, exhibits a robust association with insulin resistance in human skeletal muscle. Specifically, skeletal muscle lactylation levels are significantly elevated in obese individuals compared to lean counterparts, and this upregulation correlates positively with insulin resistance (r=0.37, p<0.05). These findings suggest that lactylation may contribute to the pathogenesis of insulin resistance by perturbing metabolic pathways, such as enhancing anaerobic metabolism and suppressing oxidative metabolism (1). Further mechanistic studies reveal that lactylation promotes IRS-1 serine 636 phosphorylation in skeletal muscle cells—a phenomenon directly linked to impaired insulin signaling cascades—thereby reinforcing its role in insulin resistance (26). Additionally, lactylation has been implicated in indirect modulation of insulin sensitivity in other bone-related disorders, potentially through regulating immune cell function and bone metabolism (27). Collectively, these discoveries position lactylation as a critical mechanistic bridge connecting lactate metabolism to insulin resistance, offering novel insights into metabolic disease pathogenesis.

#### 2.2.2 Lactylation and chronic inflammation

Lactylation has emerged as a significant regulatory mechanism in the context of chronic inflammation, particularly through its influence on macrophage polarization. Macrophages can adopt distinct phenotypes, namely M1 (pro-inflammatory) and M2 (anti-inflammatory), which are crucial for orchestrating the immune response (28). Studies have shown that lactylation can modulate the polarization of macrophages towards the M1 phenotype, thereby enhancing inflammatory responses. For instance, lactate accumulation in the microenvironment has been linked to increased levels of lactylation at specific histone residues, which in turn promotes the transcription of pro-inflammatory cytokines such as IL-6 and TNF-α. This is particularly evident in conditions such as diabetic nephropathy, where lactate exacerbates inflammation by enhancing NLRP3 inflammasome activation, leading to further tissue damage and chronic inflammatory states (7). Additionally, lactylation has been implicated in the activation of transcription factors like YY1, which regulate the expression of inflammatory genes, thereby perpetuating the inflammatory cycle (29). The interplay between lactylation and macrophage polarization highlights the potential of targeting lactylation pathways as a novel therapeutic strategy to mitigate chronic inflammation in various pathological conditions.

#### 2.2.3 Lactylation and oxidative stress

Lactylation also plays a critical role in modulating oxidative stress, a key contributor to cellular damage and disease progression (30). Elevated levels of lactate, particularly in pathological states like diabetes, have been shown to enhance lactylation of these antioxidant enzymes, thereby impairing their function and exacerbating oxidative stress (31). Furthermore, lactylation can lead to mitochondrial dysfunction, a significant source of reactive oxygen species (ROS) accumulation. For instance, studies indicate that lactylation of key mitochondrial proteins can disrupt their normal function, leading to increased ROS production and subsequent oxidative damage to cellular components (30, 31). This relationship between lactylation and oxidative stress underscores the importance of lactate as not merely a metabolic byproduct but as a critical signaling molecule that can influence both inflammatory and oxidative pathways. Targeting lactylation may thus offer a dual benefit in therapeutic strategies aimed at reducing both inflammation and oxidative stress, particularly in chronic diseases characterized by metabolic dysregulation.

### 2.3 The role of lactylation modification in diabetic complications

#### 2.3.1 Research on the molecular mechanisms of lactylation and gestational diabetes mellitus

Gestational diabetes mellitus (GDM), a common metabolic disorder during pregnancy, severely impacts maternal and fetal health. Using integrated ChIP-seq and RNA-seq analyses, a pioneering study systematically revealed, for the first time, the genome-wide distribution of histone lactylation in GDM patients and its regulatory role in gene expression (11). The study demonstrated that plasma lactate levels and histone lactylation levels are significantly elevated in GDM patients. Differentially expressed genes were primarily enriched in metabolic and signaling pathways, including PI3K-Akt, Jak-STAT, and mTOR, suggesting that lactylation may contribute to GDM pathogenesis by regulating these pathways. Notably, the CACNA2D1 gene exhibited significantly enhanced lactylation modification and upregulated expression, which promotes cell viability and proliferation, emerging as a key regulatory target of lactylation in GDM. This discovery provides novel insights into the molecular mechanisms of GDM and lays a foundation for the development of targeted therapeutic strategies.

#### 2.3.2 Multidimensional regulatory roles of lactylation in diabetic nephropathy

Diabetic nephropathy (DN), one of the most severe microvascular complications of diabetes, involves complex pathogenic mechanisms including inflammation, oxidative stress, renal fibrosis, and metabolic imbalance. In recent years, the role of lactate metabolism and its mediated lactylation modification in DN has attracted increasing attention. Lactylation exacerbates renal fibrosis via NLRP3 inflammasome activation, as previously discussed in Section 2.2.2. As a key epigenetic modification linking lactate metabolism to cell signaling, lactylation further exacerbates DN pathogenesis by impairing endothelial function, inhibiting antioxidant defense systems, and suppressing DNA repair mechanisms (7).

At the level of specific regulatory mechanisms, protein-level studies have shown that increased lactylation at the K970 site of leucyl-tRNA synthetase 1 (LARS1) promotes podocyte injury and apoptosis in DN by inhibiting autophagic activity; knockdown of LARS1 significantly improves renal function, suggesting that lactylation plays a critical role in DN by regulating protein function (3). Furthermore, lactylome studies have provided more systematic evidence for mechanistic insights: via liquid chromatography-tandem mass spectrometry (LC-MS/MS) analysis, researchers found that lactylated protein are significantly upregulated in renal tissues of diabetic mice, and lactylation at the K182 site of mitochondrial-localized acyl-CoA synthetase family member 2 (ACSF2) directly induces mitochondrial dysfunction, indicating that lactylation-mediated mitochondrial damage is an important mechanism underlying DN progression (32). Another study revealed that histone lactylation induced by lactate under high glucose conditions promotes podocyte EMT, thereby impairing glomerular filtration barrier function; meanwhile, changes in lactate transporter and metabolic enzyme activity further regulate intracellular lactylation levels, affecting the pathological state of DN (13).

In the inflammation-fibrosis regulatory axis, lactylation plays a central role. A study demonstrated that insulin-like growth factor binding protein 5 (IGFBP5) activates the NLRP3 inflammasome by regulating glycolysis-mediated histone H3K18 lactylation, thereby promoting endothelial-mesenchymal transition (EndMT) and renal fibrosis; knockdown of IGFBP5 significantly alleviates DN pathological damage, confirming the core regulatory role of lactylation in the inflammation-fibrosis cascade (33).

In summary, lactylation regulates cell injury, metabolic disorder, and fibrosis in DN through multiple targets and pathways, emerging as a potential therapeutic target for DN.

#### 2.3.3 Exploration of the role of lactylation in diabetic cognitive dysfunction

Beyond renal and retinal complications, lactylation also modulates central nervous system dysfunction in dianetes, particularly cognitive impairment.Diabetic Cognitive Dysfunction (DCD) is a critical manifestation of central nervous system impairment in diabetes mellitus, with complex and incompletely elucidated pathogenesis. In recent years, the role of lactate in brain energy metabolism has emerged as a research focus. A systematic review elaborates on the multilevel biological functions of lactate in brain energy metabolism, astrocyte-neuron lactate shuttle (ANLS), and epigenetic regulation. This review indicates that lactate can regulate the expression of neuroplasticity-related genes through histone lactylation, thereby influencing cognitive function.

The review further points out that under the pathological state of DCD, dysregulated lactate metabolism and abnormal lactylation modification may participate in the pathological process of cognitive impairment by regulating neuronal signal transduction and gene expression. Notably, the regulatory effects of lactate depend on differences in its concentration, action timing, and pathological microenvironment. Based on these findings, the review emphasizes that future studies should combine animal models and clinical observations to deeply elucidate the key target genes and signaling pathways regulated by lactylation, provding a new theoretical basis for the prevention and treatment of DCD (34).

#### 2.3.4 The role of lactylation in diabetic retinopathy

Lactylation plays a central regulatory role in the pathogenesis of diabetic retinopathy (DR). Under diabetic conditions, elevated lactate levels drive the upregulation of fat mass and obesity-associated protein (FTO) expression via histone lactylation. As an N^6^-methyladenosine (m^6^A) demethylase, FTO further enhances the stability of cyclin-dependent kinase 2 (CDK2) mRNA through an m^6^A-YTHDF2-dependent mechanism, promoting endothelial cell (EC) cycle progression and tip cell formation. This ultimately exacerbates retinal angiogenesis, vascular leakage, and inflammation, which has been validated in *in vitro* cell models, mouse, and zebrafish models (35).

Furthermore, lactylation participates in DR progression through a multidimensional epigenetic regulatory network involving crosstalk between histone lactylation, m^6^A modification, and DNA methylation. For instance, the compound Curcumol can stabilize the expression of long non-coding RNA MAFG-AS1 by activating FTO demethylase activity, thereby inhibiting high glucose-induced EC inflammation, migration, and vascular leakage (36). As an important type of post-translational modification (PTM), lactylation also aggravates diabetic endothelial dysfunction by impairing mitochondrial function, and lactylation-targeted interventions offer potential therapeutic avenues for DR management (37). Key molecules and mechanisms are summarized in **Table 1**.

**Table 1:** Key molecules and their molecular mechanisms related to lactylation modification in diabetic complications.

Diabetic complications	Key molecule	Molecular mechanism
Gestational Diabetes Mellitus	CACNA2D1	H3K18la → CACNA2D1 ↑→GDM
Diabetic Nephropathy	LARS1	LARS1 lactylation→ mTORC1 ↑→ autophagy ↓→ podocytes injury ↑→DN
Diabetic Nephropathy	ACSF2	ACSF2 lactylation →mitochondrial dysfunction →DN
Diabetic Nephropathy	Histone lactylation	Histone lactylation→ epithelial-mesenchymal transition→DN
Diabetic Nephropathy	IGFBP5	IGFBP5-H3K18la→NLRP3 inflammasome→endothelial–mesenchymal transition and renal fibrosis→DN
Diabetic Retinopathy	FTO	H3K18LA→FTO ↑→angiogenesis↑→DR

### 2.4 Current research status and future prospects

As a key molecular hub bridging cellular metabolism and gene expression regulation, lactylation has emerged as a critical mediator in metabolic regulation, inflammatory responses, cell fate determination, and tissue fibrosis associated with diabetes and its complications. However, current research faces three core challenges: First, the spatiotemporal dynamic regulatory mechanisms of lactylation remain incompletely understood, as most studies rely on *in vitro* or animal models with limited large-scale clinical validation. Second, the specific substrates and function diversity of lactylation modifications await systematic characterization, particularly regarding their differential expression patterns across distinct tissues and cell types. Third, the crosstalk between lactylation and other epigenetic modifications (e.g., methylation, acetylation), along with their synergistic effects on gene regulatory networks, remain to be fully elucidated.

Future investigations should prioritize five directions: (1) Leveraging multi-omics integration technologies to dynamically profile the spatiotemporal changes in lactylation expression across distinct pathological stages and tissues, aiming to clarify its regulatory thresholds and temporal dynamics; (2) Conducting in-depth mechanistic analysis of key lactylation target genes and their downstream signaling cascades to unravel the specific mechanistic roles in metabolic reprogramming, inflammation, and fibrosis; (3) Developing lactylation-specific small-molecule inhibitors or modulators to evaluate their therapeutic potential in diabetes and its complications; (4) Validating the clinical utility of lactylation as a biomarker and therapeutic target (including efficacy and safety assessments) via integrated studies of clinical samples and preclinical animal models; (5) Expanding mechanistic investigations into lactylation in diabetic cognitive dysfunction to facilitate the comprehensive management of neuro-metabolic disorders.

In summary, lactylation holds substantial promise for both research advancement and clinical translation. Future interdisciplinary investigations will deepen our mechanistic insights into lactylation, thereby paving the way for the development of novel precision diagnostic and therapeutic strategies for diabetes and its complications.

## 3 Conclusion

Lactylation linking metabolism to epigenetic regulation, is a pivotal molecular node in the pathophysiology of diabetes and its complications. It plays dual roles as a metabolic sensor and transcriptional modulator, regulating gene networks for metabolic homeostasis (e.g., insulin signaling via IRS-1) and disease progression (e.g., NLRP3 inflammasome in fibrosis) through histone/non-histone modifications. Tissue-specific regulatory patterns are observed: in diabetic nephropathy, it promotes podocyte injury (LARS1 lactylation) and mitochondrial dysfunction (ACSF2 modification); in retinopathy, the lactylation-FTO-CDK2 axis drives pathological angiogenesis; in gestational diabetes, histone lactylation dysregulates PI3K-Akt signaling. Despite progress, three critical challenges remain: unclear spatiotemporal dynamics in clinical settings, uncharacterized substrate specificity/functional diversity across cell types, and elusive crosstalk with other epigenetic modifications (e.g., acetylation). Future research should prioritize longitudinal clinical validation, identification of tissue-specific substrates, and development of selective modulators to advance precision therapies targeting the metabolic0epigenetic interface in diabetes.
